# Effect of Tilt-in-Space and Reclining Angles of Wheelchairs on Normal Force and Shear Force in the Gluteal Region

**DOI:** 10.3390/ijerph19095299

**Published:** 2022-04-27

**Authors:** Hitoshi Koda, Yohei Okada, Takahiko Fukumoto, Shu Morioka

**Affiliations:** 1 Department of Rehabilitation Sciences, Faculty of Allied Health Sciences, Kansai University of Welfare Sciences, 3-11-1, Asahigaoka, Kashiwara 582-0026, Osaka, Japan; 2 Department of Physical Therapy, Faculty of Health Science, Kio University, 4-2-2, Umami-naka, Kitakatsuragi-gun, Koryo-cho 635-0832, Nara, Japan; y.okada@kio.ac.jp (Y.O.); t.fukumoto@kio.ac.jp (T.F.); s.morioka@kio.ac.jp (S.M.)

**Keywords:** normal force, shear force, tilt-in-space, reclining, wheelchair

## Abstract

Healthcare workers need to educate patients regarding proper sitting positions to prevent pressure injuries in the elderly and disabled. The purpose of this study was to investigate the differences in normal and shear force in the gluteal region using the combination of tilt-in-space and reclining functions of wheelchairs. Twelve healthy subjects were recruited. Protocols for 15 wheelchair tilt-in-space and reclining angles, including three reclining angles (0°, 10°, and 20°) and five tilt-in-space (0°, 5°, 10°, 15°, and 20°), were randomly assigned. To measure the amount of normal and shear force applied to the gluteal region while sitting on a wheelchair, a force plate was placed on the seat to measure the seat reaction force. For statistical analysis, a two-factor analysis of variance, with tilt-in-space and reclining, was performed for each normal and shear force. The normal force showed a significant decrease with increased reclining angle. For the shear force combined with sagittal and lateral components, the 10° tilt-in-space showed a significant decrease compared to other conditions. The combination of 20° reclining and 10° tilt-in-space angles may decrease both normal and shear force in the gluteal region while sitting. These findings may help wheelchair-dependent individuals avoid pressure injuries.

## 1. Introduction

Wheelchairs are used to prevent elderly and disabled people from becoming bedridden [1]. However, for people with disabilities, prolonged sitting prevents movements such as postural changes and causes pressure injuries. Treatment for pressure injuries requires a long period of bed rest, resulting in economic and psychological burdens for the patient and the caregiver [2,3,4]. Four local factors contribute to generating pressure injuries: pressure force, shear force, temperature, and moisture [5]. Additionally, Bluestein and Javaheri (2008) reported that predisposing factors for pressure injuries include poor nutrition, aging skin, and friction [6]. Although the main management of pressure injuries is nursing care, appropriate rehabilitation is vital to reducing mechanical forces when becoming mobile. Particularly, healthcare workers often educate patients regarding proper sitting positions in the wheelchair and positioning on the bed to reduce normal and shearing force [7]. In recent years, many studies have been conducted on human sitting posture, including sitting comfort and the load placed on the body [8,9].

Commonly used strategies for preventing pressure injuries include using high-quality wheelchair seat cushions to reduce normal force by improving pressure distribution [10]. Previous studies have reported that normal force cannot be ignored as a contributing cause of pressure injuries [11,12]. However, various studies have strongly suggested that pressure reduction was only one of several other factors, such as friction and shear force [13,14,15]. Linder and Gefen (2007) demonstrated that skeletal muscle tissue of rats can survive 2 h of complete ischemia but have been shown to die within 15 min when a load causes tissue shear deformation [14]. Sonenblum and Sprigle (2011) reported that pressure force was not reduced even with increased blood flow with a wheelchair adjustment. Thus, shear force in the sitting position needs investigation [15].

The tilt-in-space and reclining functions of wheelchairs are responsible for the normal or shear force relief and increased blood flow to the tissue previously deprived of oxygen [16,17]. Ding et al. (2008) reported that tilt-in-space significantly reduced pressure force, a key component in pressure injury development, and that combining tilt-in-space with backrest recline reduced pressure more than tilt-in-space alone [16]. Zemp et al. (2019) demonstrated that small tilt-in-space and recline angles can reduce sitting interface pressures and that changes in ischial blood flow only occur at larger angles [17]. However, to the best of our knowledge, few studies have investigated the effects of tilt-in-space and reclining functions in wheelchairs on shear force. Therefore, it is necessary to clarify both normal and shear force for each wheelchair sitting position.

## 2. Objective

This study aimed to investigate the difference in normal and shear forces in the gluteal region using the combination of tilt-in-space and reclining functions in wheelchairs.

## 3. Materials and Methods

### 3.1. Participants

This study recruited 12 healthy subjects (6 men and 6 women). The mean age was 22.5 ± 1.6 years, mean height was 168.0 ± 8.7 cm, and mean body mass was 59.6 ± 7.9 kg. No participants had a significant past medical history.

The study was performed in accordance with the World Medical Association’s Declaration of Helsinki. The purpose, nature, and potential risks of the experiments were fully explained to the participants, and all participants provided written informed consent prior to their inclusion in the study. The study protocol was approved by the research ethics committees of the respective affiliated institutes (H23-21).

### 3.2. Testing Procedures and Protocol

We used a wheelchair (back height: 89.5 cm, seat depth: 42.0 cm) with reclining and tilt-in-space functions. This wheelchair was selected because of its passive reclining and tilt-in-space design, which is typically used for the elderly or individuals with severe physical disabilities. A 15-mm-thick wooden board was placed on the surface of the seat and backrest to prevent bending. To measure the seat reaction force to determine the amount of normal and shear force applied to the gluteal region while sitting, a force plate (MG-100, ANIMA, Tokyo, Japan) was used [18]. The vertical component was the normal force, and the sagittal and lateral components were the shear force. Therefore, the force plate could measure the component of the force in each direction based on the force applied to the sensor located at each corner of the plate.

**Figure 1:**
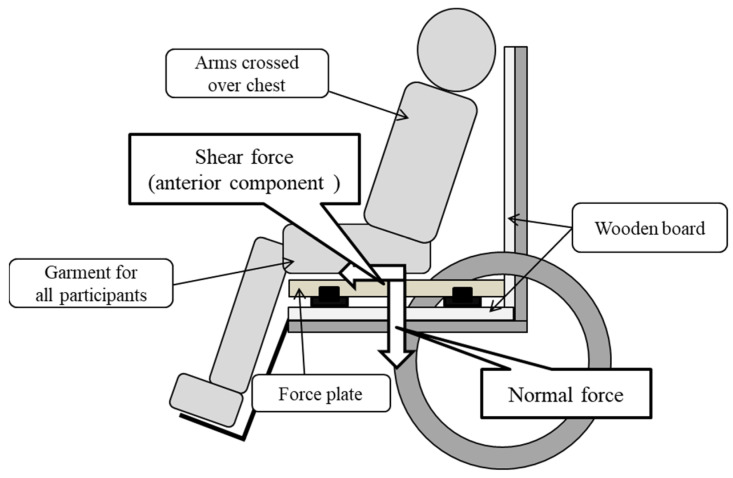
Measuring method. Participants sat on the force plate and were placed in a relaxed sitting position.

Participants were placed in a relaxed sitting position on the force plate with slight hip abduction and were instructed not to change their posture during the measurement (Figure 1). The placement of the buttocks in the sitting position was 5 cm in front of the deepest sitting position; this was considered to be a comfortable sitting posture. The arm support was not used, and the arms were folded at the chest. The height of the foot support was adjusted so that the long axis of the thigh and seat surface were horizontal. Each subject was provided with a pair of soft, comfortable pants without rear pockets to standardize clothing while seated.

All subjects performed 15 different wheelchair seating positions with three reclining angles (0°, 10°, 20°) and five tilt-in-space angles (0°, 5°, 10°, 15°, 20°). The order of the 15 conditions was determined by a random number table for each subject. The sitting conditions totaled 15 wheelchair configuration comparisons; these conditions were based on the frequency of wheelchair angle usage in patients with severe disabilities [14]. Participants maintained the sitting position on the wheelchair. We provided a minimum of 2 min settling time under each condition and measured the seat reaction force for 3 min. Data were collected at a 20-Hz sampling rate. The average values of the normal force, the sagittal component of the shear force, the lateral component of the shear force, and the shear force combined with the sagittal and lateral components were calculated using trigonometric methods. These values were different depending on the body weight of the subject; thus, body weight percentages were calculated and used to determine the normal and shear force on the buttocks [19].

### 3.3. Statistical Analysis

The analysis of variance of two factors, tilt and reclining, was performed for each normal and shear force using a two-way repeated-measure analysis of variance (ANOVA). When the main effect of reclining or tilt-in-space was shown, a multiple comparison test using the Bonferroni method was performed to examine the difference in angle. When an interaction was observed, a simple main effect test (one-way ANOVA and Bonferroni’s multiple comparison test for each condition) was performed to verify whether the obtained main effect was observed for each condition. For example, if an interaction was observed and the main effect in the tilt-in-space was observed, then the five tilt-in-space conditions were compared for each reclining angle. SPSS software for Windows version 24.0 (IBM, Armonk, NY, USA) was used for the data analysis. The level of statistical significance was set at *p* < 0.05.

## 4. Results

**Figure 2:**
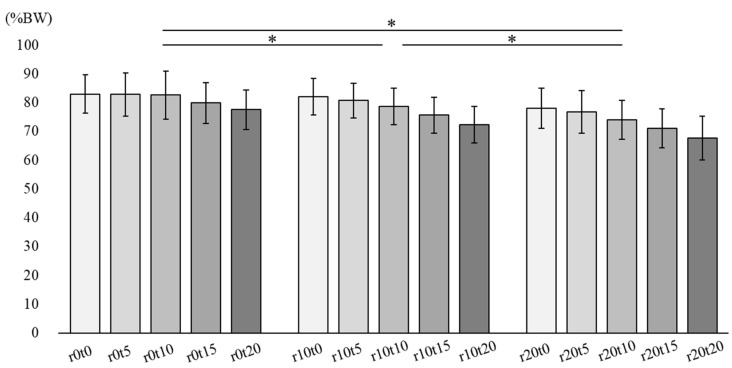
Normal Force. R: reclining, t: tilt-in-space. * There were significant differences of reclining condition in multiple comparisons.

Figure 2 describes the normal force in 15 sitting conditions. There was no interaction between tilt-in-space and reclining. The main effect was observed during reclining (*p* < 0.01). In the multiple comparison analysis, reclining at 10° and 20° showed a significant decrease when compared to the reclining angle of 0° in normal force. Reclining at 20° showed a significant decrease when compared to reclining at 10° (*p* < 0.01).

**Figure 3:**
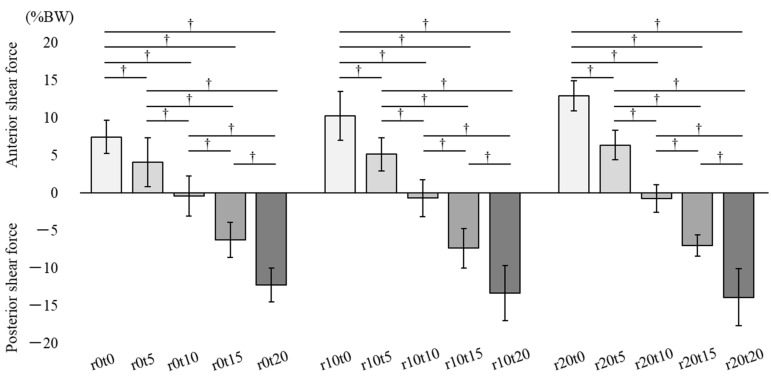
Sagittal component of the shear force. The positive value in the sagittal component represents the anterior shear force that occurs on the buttocks, and the negative value represents the posterior shear force. r: reclining, t: tilt-in-space. † There were significant differences of tilt-in-space condition in the simple main effect test.

Figure 3 describes the sagittal component of shear force in 15 sitting conditions. The positive value represents the anterior shear force occurring on the buttocks, and the negative value represents the posterior shear force. There was an interaction between tilt-in-space and reclining, and the main effect was observed in tilt-in-space (*p* < 0.01). In the simple main effect test, there were significant differences between each tilt-in-space condition in combination with reclining angles of 0°, 10°, and 20°, respectively. The anterior component of the seat reaction force in the 5° and 10° tilt-in-spaces significantly decreased, and the posterior component in the 15° and 20° tilt-in-spaces significantly increased compared to those in the 0° tilt-in-space. The anterior component in the 10° tilt-in-space significantly decreased, and the posterior component in the 15° and 20° tilt-in-spaces significantly increased compared to those in the 5° tilt-in-space. The posterior component in the 15° and 20° tilt-in-spaces significantly increased compared to that in the 10° tilt-in-space, and the posterior component in the 20° tilt-in-space significantly increased compared to that in the 15° tilt-in-space (*p* < 0.01).

**Figure 4:**
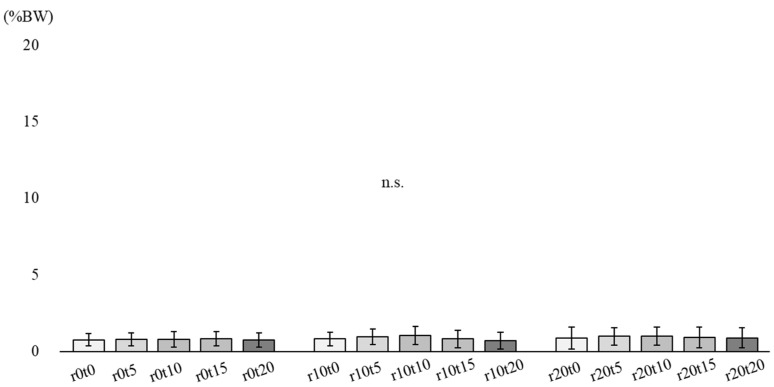
Lateral component of the shear force. R: reclining, t: tilt-in-space, n.s.: non-significant.

Figure 4 describes the lateral component of shear force in 15 sitting conditions. There was no interaction between tilt and reclining. No main effect was observed in either tilt-in-space or reclining.

**Figure 5:**
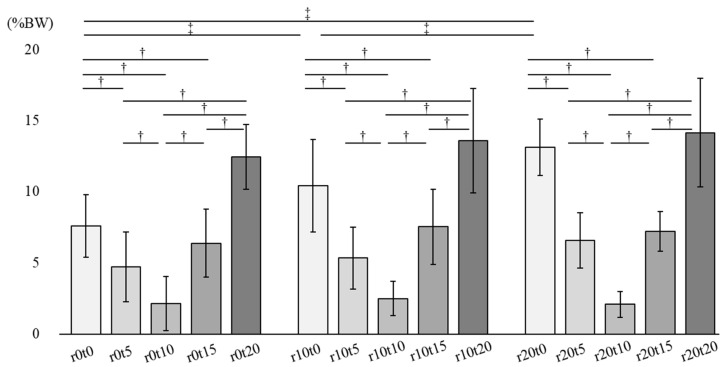
Shear force combined with sagittal and lateral components. R: reclining, t: tilt-in-space. † There were significant differences of tilt-in-space condition in the simple main effect test. ‡ There were significant differences of reclining condition in the simple main effect test.

Figure 5 describes the shear force combined with sagittal and lateral components in 15 sitting conditions (*p* < 0.01). There was an interaction between tilt and reclining, and the main effect was observed in tilt-in-space and reclining (*p* < 0.01). In the simple main effect test, there were significant differences between each tilt-in-space condition in combination with the reclining angles of 0°, 10°, and 20°. The shear force in the 10° tilt-in-space significantly decreased compared to that in the 0°, 5°,15°, and 20° tilt-in-spaces. The shear force in the 5° and 15° tilt-in-spaces significantly decreased compared to that in the 0° and 20° tilt-in-spaces. Additionally, there were significant differences between each reclining condition only in combination with tilt-in-space angles of 0° in the simple main effect test. The shear force in the 10° and 20° reclining significantly increased compared to that in the 0° reclining. The shear force in the 20° reclining significantly increased compared to that in the 10° reclining in combination with tilt-in-space angles of 0° (*p* < 0.01).

## 5. Discussion

In the present study, we investigated the difference in normal and shear force in the gluteal region using the combination of tilt-in-space and the reclining function of wheelchairs. The normal force showed a significant decrease as the reclining angle increased. A reduction in the forward shear force and an increase in the backward shear force were observed as the tilt-in-space angle increased. There was no significant difference in the lateral component of the shear force between tilt and reclining. For the shear force combined with sagittal and lateral components, the 10° tilt-in-space showed a significant decrease when compared to other conditions.

Fu et al. (2014) demonstrated that, in a comfortable sitting position, pelvic posterior leaning occurred with wheelchair reclining [20]. Posterior leaning of the pelvis by wheelchair reclining may disperse the normal force applied to the buttocks and thighs to the back support [21]. In our study, the normal force showed a significant decrease as the reclining angle increased. Therefore, even when used in combination with tilt-in-space, wheelchair reclining played a role in redistributing the force applied to the back support and in reducing the normal force.

The sagittal component of the shear force showed an interaction with tilt-in-space and reclining, but the main effect was found only in tilt-in-space. Furthermore, the sagittal component of the shear force at the 10° tilt-in-space was the lowest for each reclining combination. In a comfortable sitting position in a wheelchair, individuals lean on the back support to augment the base of support and to obtain stability [16]. The reaction force in the anterior direction on the buttocks was created by contact with the back support [22]. The tilt-in-space function is the inclination of the seat, and the sliding force in the posterior direction was created by increasing the tilt angle [23]. It is possible that the reaction force in the anterior direction and posterior sliding force in the 10° tilt-in-space were almost equal, leading to the lowest shear force in the 10° tilt-in-space. In the 15° or 20° tilt-in-space, the posterior sliding force might have been larger than the reaction force in the anterior direction, and there was a posterior shear force. Therefore, tilt-in-space could have decreased the shear force in the anterior direction, and more tilt-in-space might have increased the shear force in the posterior direction.

The lateral component of the shear force showed no interaction or main effects. Furthermore, the lateral component of the shear force was low in value, and the value of the shear force combined with the sagittal and lateral components was close to the value of the sagittal component of the shear force. Therefore, if the patient does not have scoliosis or hemiplegia, the influence of the lateral component of the shear force may not be a concern.

The shear force combined with the sagittal and lateral components increased significantly as the reclining angle increased only in combination with tilt-in-space angles of 0°. Conversely, the normal force decreased significantly as the reclining angle increased (Jan et al., 2010). However, without the combination with tilt-in-space, reclining causes the shear force to increase. Furthermore, the shear force combined with sagittal and lateral components at the 10° tilt-in-space was the lowest for each reclining combination. Therefore, even when used in combination with reclining, the 10° tilt-in-space of the wheelchair may play a role in decreasing shear force to the gluteal region.

This study had several limitations. First, this study was a pilot study on healthy adults, and we did not measure posture or alignment. The results might have varied if we had used elderly patients with scoliosis and patients with spinal cord injury. Second, we measured only for 3 min under each condition. We measured the seat reaction force in the sitting position to determine how the forces change in a preliminary study and confirmed that the value after 2 min was stable. Although it is not possible to be certain that there were changes in the seat reaction force over time, the results of this study may apply to various cases. In future studies, we will investigate the posture of participants and the effect of sitting for over an hour in a wheelchair. Third, the condition of sitting on the force plate and wooden boards was not common in terms of plate size or hardness. We also could not consider the force on the user’s back. We should use embedded sensors or flexible pressure films on the buttock and back for increasing the accuracy and minimizing the interference with normal sitting in a wheelchair in future studies.

## 6. Conclusions

This study investigated the difference in normal and shear force in the gluteal region using the combination of tilt-in-space and reclining functions of wheelchairs. The results indicate that the normal force showed a significant decrease with increased reclining angle, and the 10° tilt-in-space decreased significantly in shear force when compared to other conditions. Thus, the combination of a 20° reclining angle and a 10° tilt-in-space angle decreases both the normal and shear force in the gluteal region while sitting on a wheelchair.

## Data Availability

The Supplementary Material is available in the electronic version of this article.

## Supplementary Materials

The following supporting information can be downloaded at: https://www.mdpi.com/article/10.3390/ijerph19095299/s1.
